# Stakeholders Opinions on Multi-Use Deep Water Offshore Platform in Hsiao-Liu-Chiu, Taiwan

**DOI:** 10.3390/ijerph15020281

**Published:** 2018-02-06

**Authors:** Ya-Tsune Sie, Pierre-Alexandre Château, Yang-Chi Chang, Shiau-Yun Lu

**Affiliations:** 1 Department of Marine Environment and Engineering, National Sun Yat-sen University, Kaohsiung 80424, Taiwan; angelshieh59@gmail.com (Y.-T.S.); shiauyun@faculty.nsysu.edu.tw (S.-Y.L.); 2 Department of Bioenvironmental Systems Engineering, National Taiwan University, Taipei 10617, Taiwan

**Keywords:** offshore platform, group model building, stakeholders, causal-loop diagram, systems thinking

## Abstract

This paper describes a group model building activity designed to elicit the potential effects a projected multi-use deep water offshore platform may have on its local environment, including ecological and socio-economic issues. As such a platform is proposed for construction around the island of Hsiao-Liu-Chiu, Taiwan, we organized several meetings with the local stakeholders and structured the debates using group modeling methods to promote consensus. During the process, the participants iteratively built and revised a causal-loop diagram that summarizes their opinions. Overall, local stakeholders concluded that a multi-use deep water offshore marine platform might have beneficial effects for Hsiao-Liu-Chiu because more tourists and fish could be attracted by the structure, but they also raised some potential problems regarding the law in Taiwan and the design of the offshore platform, especially its resistance to extreme weather. We report the method used and the main results and insights gained during the process.

## 1. Introduction

**Figure 1:**
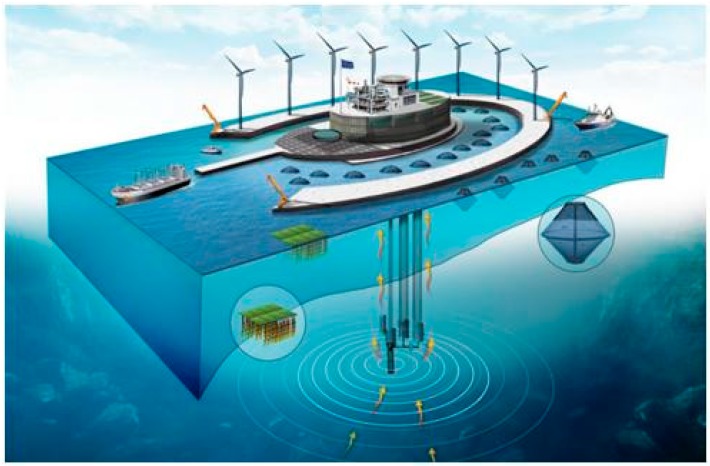
Green and Blue Concept (Fernando Montecruz for the Modular Multi-use Deep Water Offshore Platform Harnessing and Servicing Mediterranean, Subtropical, and Tropical Marine and Maritime Resources (TROPOS) project. (http://www.troposplatform.eu)).

Increasingly limited land and resources worldwide have steered global attention toward applying and developing marine resources such as ocean energy. To ensure marine resource sustainability and prevent damages to the marine environment, the European Union has actively sought to formulate integrated strategies, action plans, and technologies for the sustainable use of marine resources. The European Union, under the Ocean of Tomorrow call proposed in the Seventh Framework Programme, approved the “Modular Multi-use Deep Water Offshore Platform Harnessing and Servicing Mediterranean, Subtropical, and Tropical Marine and Maritime Resources” project (TROPOS, project ID: 288192) to develop floating multi-use platforms (MUP) capable of operating in deep water, where fixed structures are not feasible. In addition to the modular designs of offshore platforms, TROPOS addresses the potential social, economic, and environmental impacts of the projects, particularly the potential benefits and risks involved in using certain marine resources. Such a comprehensive assessment is mandatory for the future design and development of multi-purpose offshore platforms. In TROPOS, the Hsiao-Liu-Chiu island (later referred to as Liu-Chiu) in Taiwan was selected for developing a green and blue platform that provides self-sufficient sea ranching through integrating marine energy and aquaculture (Figure 1). The multipurpose offshore platform, which is proposed for construction off the Taiwanese coast, would provide energy through ocean thermal energy conversion (OTEC), the requirement of which is fulfilled by the near shore water depth of Liu-Chiu. The platform also enables developing local cage aquaculture and could be suitable for tourist and recreational activities such as recreational fishing and seafood dining.

OTEC primarily involves utilizing the temperature difference between surface and deep ocean water, which is due to the ocean absorbing solar energy. The difference in temperature alternately condenses and evaporates a working fluid, generating water volume and pressure charges that can rotate turbines to drive generators and produce electricity [1]. Situated next to the Kaoping submarine canyon, Liu-Chiu offers a suitable environment for OTEC technology. The temperature difference between the surface and the deep layer at the Kaoping Canyon site is 15 °C at 300 m depth. OTEC generates no carbon dioxide, and the mixing of surface and deep waters facilitates the growth of phytoplankton on the ocean surface, which consumes carbon dioxide. OTEC may also provide other economic benefits: processed water can be used for aquaculture, irrigation, drinking, hydrogen-producing electrolysis, and refining trace elements or minerals [2,3]. However, in OTEC, energy is stored in the form of ammonia and methanol, which may damage the marine environment if leaked. Additionally, thermal energy conversion could alter the water temperature, microorganisms may be sucked into pipelines, and installing OTEC equipment might damage marine habitats [4].

Shortage of land and water resources has hindered the sustainable development of conventional coastal aquaculture in Taiwan, and excessive ground water harvesting has caused subsidence and land salinization, incurring substantial costs on Taiwanese society [5]. Cage aquaculture generates a similar yield as coastal aquaculture does [6] and requires no land, freshwater resource, or pumping ventilation facility. Therefore, cage aquaculture has become a viable option for developing aquaculture in Taiwan. However, cage aquaculture also incurs environmental impacts [7]. Unconsumed feed and fish waste may accumulate on the seabed, increasing the concentration of sedimentary organic matter, which consumes oxygen in the bottom materials around cages and subsequently causes an oxygen deficiency [8]. Oxygen depletion causes the release of nitrogen, hydrogen sulfide, and methane from the sediment, jeopardizing marine life. Additionally, upwelling bottom ocean water with low dissolved oxygen may increase fish mortality in the upper ocean. Typically, cage aquaculture affects benthos within 20–50 m. In some areas, mismanagement and abnormal sea conditions have expanded the range of impact to 150 m [9,10]. For example, in Taiwan, scientists have reported impacts of up to 500 m away from aquaculture cages during the monsoon season in the Bay of Magong, Penghu [11].

Constructing, operating, and removing offshore platforms can affect coastal residents and marine environments. Noise, light, and dust pollution reduce the quality of residential life. Piles may collide directly with marine organisms when driven, and cables and foundation piles may affect benthos or cause sediment re-suspension, raising seawater turbidity and undermining the net primary production of oceans [12,13,14]. Moreover, waste from construction, transportation, and machinery can damage reefs and other marine organisms, impairing the marine environment [15]. Furthermore, waste products affect coastal areas directly and may contain toxic materials, which can cause health problems for residents. Such waste problems could be exacerbated by platform removal processes if waste disposal regulations are not enforced.

If the offshore platform is constructed in Liu-Chiu in accordance with the TROPOS project, then it will have multifaceted impacts on local industries and stakeholders, such as the relationship between cage aquaculture and local fisheries, local sea use, and the island of Liu-Chiu residents. Conventional interviews and questionnaire surveys have been conducted to study local residents’ and tourists’ social acceptance of the offshore platform. Results indicate that the majority of local residents stated that they would oppose the development of the platform, whereas the majority of tourists would support it, provided that the platform offers leisure and renewable energy features [16].

Typically, interviewees have expressed their opinions through brief discourses or by discussing the extent of the influence that the platform will have according to one particular dimension. However, the construction of the multi-purpose offshore platform will affect Liu-Chiu in multiple dimensions, the interrelations among which are considerable. It is therefore very unlikely that respondents were able to deliver a well-thought answer to such complex issue without time to reflect on it. As a consequence, and in order to complete the surveys, we designed a Group Model Building (GMB) initiative to better understand the mental models of local stakeholders. A group model requires the repeated participation of internal group members for a comprehensive analysis of the system under study. Internal members are typically system stakeholders who understand the system structure. Therefore, the model building process requires guiding the stakeholders to discuss and present their abstract opinions, expected system behavior, and influential factors in a macro-system structure through systems thinking, thereby enabling decision-makers to analyze the management strategies effectively [17,18,19,20,21,22].

We investigated the opinions and visions of local stakeholders regarding the offshore platform. We explained the purposes, location, and design of the offshore platform to stakeholders and formulated a model based on their thoughts and opinions, which are illustrated in a Causal Loop Diagram (CLD) depicting the effects of the offshore platform on the development of the island. Subsequently, we explored the potential problems caused by the platform and potential solutions to provide feedbacks to the decision-makers and designers of the platform project. Before executing any project, relevant stakeholders should be permitted to partake in making critical decisions in advance to avoid irreversible environmental, social, and economic damages. In addition, public protests can be prevented, thereby minimizing potential reparation costs from civil mediation and reconstruction.

## 2. Method

We adopted GMB and systems thinking to elicit the opinions and visions of stakeholders and formulate their underlying mental model. Influential factors indicated by the stakeholders as affecting Liu-Chiu were organized into a Causal-Loop Diagram (CLD) to comprehensively illustrate how they interact to form the system structure.

Systems thinking emerged from von Bertalanffy’s systems theory [23] and was further developed by Forrester [24], who promoted the idea that an engineering-like approach could be adopted when examining social systems. Unlike conventional analysis methods for investigating individual system components, the systems thinking approach involves examining the behavior derived from interactions among the components, particularly when complex dynamic problems are considered [20,24].

The systems thinking approach was developed to ameliorate the flaws of conventional management science. Rather than thinking linearly or concentrating on individual cases, systems thinking emphasizes placing a problem in its environment and incorporating the concepts of causal loops and delays to identify the cyclical causal relationships and dynamics of the problem. Furthermore, it enables observers to clarify behavioral structures hidden in complex situations as well as delays in causal relationships, thus effectively distinguishing between leverage and symptomatic solutions [21,25,26,27,28,29].

**Figure 2:**
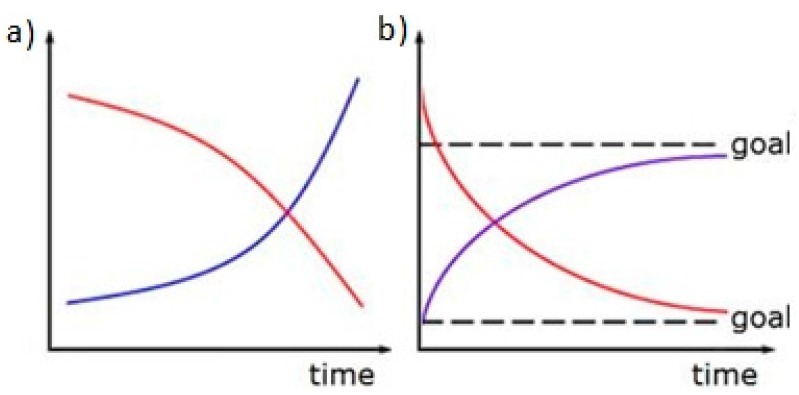
Temporal sequence charts of positive (**a**) and negative (**b**) feedback loops.

The structure of a CLD comprises a network of numerous causal links (represented by arrows), which indicate the positive and negative relations among variables. Positive causal links (“+”) indicate that a directional change in a certain variable induces a change in the same direction for another variable. On the contrary, negative causal links (“−”) depict changes in the opposite direction. When a loop has zero or an even number of negative links, it is a positive feedback loop; conversely, when a loop has an odd number of negative links, it is a negative feedback loop [30]. A positive feedback loop has a cumulative effect on a system, generating a continuous (exponential) growth or rapid deterioration. Figure 2a shows that such effects can be positive or negative. By contrast, a negative feedback loop has a moderating effect, which prompts a system to adjust and correct itself until a goal is reached. In other words, the difference between the actual situation and the goal causes the system to self-adjust to ensure that goals are achieved (Figure 2b).

Finally, delays reveal the temporal difference between the action and its result, thus impairing the ability of actors to immediately identify the effects of their action [25]. These three components were employed to draw a CLD depicting the interactions among the influential factors and to clarify the complex and dynamic system structure.

System dynamics (SD) takes the systems thinking approach further towards numerical modeling and dynamic simulation of complex systems [31]. Since its emergence in the 1950s with the work of Jay Forrester [24], SD has since been applied to a wide range of topics, especially those dealing with long-term decision-making such as in natural resources modeling [32,33,34]. Local stakeholders were originally consulted as data providers, but they are now more and more involved in the modeling process as system designers [35]. This implication in the modeling process, referred to as GMB, seeks to strengthen the learning capability of a group with the goals of reaching consensus within the group and support decision-making by group members. GMB requires a facilitator to collaborate with the participants in building the model. Because the knowledge needed for building the model is already available in the participants’ minds, mutual participation is necessary for a mental model to be extracted [36,37,38,39,40]. Mental models reveal the effect of the sub consciousness on understanding the surrounding environment and the assumptions, prejudices, or impressions regarding how to take an action. People are seldom aware of their own mental models and their effects on their actions [25]. Participating in GMB enables members to reflect on the structure of their own mental model, thereby facilitating model development and support [41,42,43].

When stakeholders participate in a discussion, their opinions and information about the system must be organized and guided. Therefore, information guidance and convergence techniques must be applied to effectively extract information [17,18,19,30,44]. As with any modeling exercise, GMB can be divided into five steps [30,31,45]. The first two steps concern system conceptualization and involve identifying the characteristics of a problem over time, and how this behavior is produced by the system structure. The remaining three steps are more technical and follow the formation of the network of causal relationships to develop a quantitative SD model, the evaluation and verification of model behavior and the analysis of various management policies [30,31,39]. These steps are detailed as follows:

(1) Problem Articulation

To understand the characteristics of a system, its problems, and the goal and range of the model, the timeframe must first be defined and all factors affecting the problems must be explained. Additionally, the hierarchy of the core topics must be clarified and repeatedly corrected through convergent and divergent tasks to identify the main issues with suitable breadth and depth in the model, and to derive their indices and quantified units.

(2) Dynamic Hypotheses

Dynamic hypotheses are tested to further identify the causal relationships among the system factors. Participants in the model-building process express their opinions on the core topics and define potentially desirable and undesirable system behaviors accordingly. Positive and negative feedback loops are then combined to visualize the structure of the system in the form of a CLD.

(3) Model Building

The CLD, which conceptualizes the system structure, is converted into a quantifiable SD model. A stock-flow diagram is formulated to simulate the system.

(4) Model Verification

The reliability of the SD model, including its structural and behavioral validity, is checked. If errors are identified, then the process returns to Step 3 for the model to be revised.

(5) Scenario Analysis

Once the SD model is verified, it can be applied to assess system behavioral changes in various scenarios. In addition, stakeholders can discuss and understand the difference between the system simulation results and their initial opinions toward the topics and reflect and improve their mental model, thus achieving consensus on the description of the system.

Because the TROPOS project is at a very early stage of development in Liu-Chiu, uncertainties related to the platform (e.g., structure, internal design, location, and regulations) prevented the collection of data. Consequently, we were unable to thoroughly apply the GMB process to develop a working SD model for quantitative simulation. Nevertheless, we still upheld the principles of GMB and systems thinking, in executing the first two steps of GMB along with the stakeholders. Finally, a conceptual CLD was built with them to illustrate their visions concerning the multipurpose offshore platform. Although a CLD cannot be used for quantitative simulation, it nevertheless depicts system structure and can therefore be used to support the discussion on potential impacts of the offshore platform on Liu-Chiu and policy analysis.

## 3. Group Model Building

The stakeholders participating in this study were the township chief of Liu-Chiu, local cage aquaculture workers, and the chair of the Liu-Chiu station of the Dapeng Bay National Scenic Area (NSA). As experts on the local tourism industry, usage of the surrounding waters, and local fisheries, the stakeholders contributed a considerable amount of information to the model building process. Meaningful indices were compiled according to their knowledge of the core topics related to the system. The relationship between the dynamic hypotheses and the system structure was then determined and presented in a CLD. The aforementioned GMB process was divided into five meetings, each of which lasted for 1 to 2 h over a period of 4 months. Table 1 depicts the GMB procedure.

The first meeting involved explaining the project to the stakeholders and determining whether they would be willing to participate in the study. The stakeholders participating to the first meeting were the township chief of Liu-Chiu, local cage aquaculture workers, the chair of the Liu-Chiu station of the Dapeng Bay NSA and the director general of the Liu-Chiu fishermen’s association. The participants agreed to participate in the study and provide relevant information, but they were dissatisfied with the location of the offshore platform as planned in the TROPOS proposal for Taiwan. The director general of the Liu-Chiu fishermen’s association strongly disapproved of the construction of the platform because of the negative impact it would have on local fisheries, and declined to participate further in the study.

**Figure 3:**
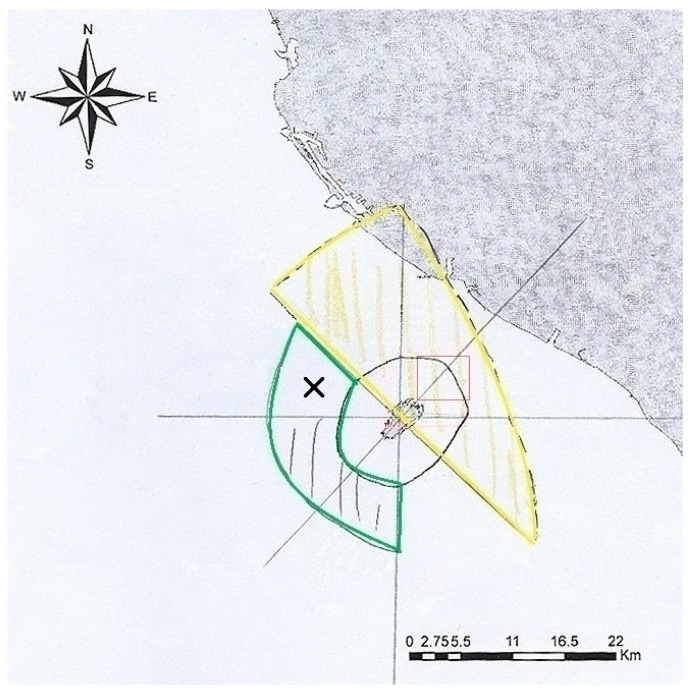
Location of the offshore platform, as proposed by the stakeholders (black cross: proposed location, black circle: 3 nautical miles radius, red square: initially proposed location, green area: suitable area for OTEC (ocean thermal energy conversion), yellow area: fishing grounds).

The second meeting began with a discussion on selecting a new location for the platform, and the stakeholders were asked to select what they considered to be the most desirable location. The stakeholders indicated that the location originally determined in the TROPOS proposal (red square on Figure 3) would impair daily transportation between Liu-Chiu and the main island of Taiwan, as well as local fisheries. Therefore, a location northwest of Liu-Chiu was selected as a more suitable location for the platform (black cross on Figure 3). The sea depth at the new location is suitable for OTEC (green area) and there would be considerably less interference with transportation and local fisheries (yellow area). Moreover, an artificial reef has already been installed there to restore the tuna population. Additionally, it was argued that the strong water current to the southwest of Liu-Chiu would reduce the likelihood that residual feed from the cage aquaculture would sediment at the bottom. Following the selection of a new location for the platform, the core topics regarding the platform construction were discussed. Adopting the nominal group technique [46], the stakeholders individually listed the topics they considered relevant to the platform without discussing their opinions with each other (Table 2). Five of these topics, namely ecology, tourism, economy, regulations, and safety, were voted as the most crucial topics. The variables affecting each of the selected topics were then discussed, and the key variables were determined and marked with stars as illustrated in Table 3. The stakeholders stated their belief that constructing the offshore platform would have a minor effect on the coastal ecosystem of Liu-Chiu and attract more tourists. In addition to the cage aquaculture, the OTEC-processed deep ocean water could stimulate Liu-Chiu’s economy. Furthermore, the stakeholders were concerned about the jurisdiction for the platform and the feasibility of its construction. Investigating these concerns revealed that there is currently only one offshore platform in Taiwan—an aquaculture facility located in Penghu—and it is under the jurisdiction of the Penghu county government. Moreover, that type of platform differs from the one in this study. Therefore, in the model-building process, it was assumed that the platform can be constructed, although the legal feasibility of the project was not examined. Regarding safety, the stakeholders feared that the platform structure might not be typhoon resistant; in the event that wreckage is carried to the coast of Liu-Chiu, the coral ecosystem there would be damaged. However, it was thought that this technical issue was beyond the scope of this study and thus was not considered a factor in the GMB process. A preliminary CLD was constructed according to the information derived from the second meeting and discussed at the subsequent meetings.

The third meeting involved verifying the consistency of the CLD with the opinions of the stakeholders. According to the topics selected at the preceding meeting, the CLD was divided into five subsystems: ecology, tourism, economy, regulations, and safety. The stakeholders indicated that discussing only the fish populations in the area oversimplified the ecological community of Liu-Chiu and that corals should be included as an ecological subsystem. In addition, other variables were added to describe the tourism–ecology relationship. Attracting more tourists not only affects the ecology, but also places further burden on the service capacity of the Liu-Chiu tourism industry. Therefore, the service equipment and facilities on Liu-Chiu were included as a factor in the tourist subsystem. The stakeholders expressed that the regulation subsystem inadequately addressed their concerns and required revisions. Some parts of the preliminary CLD did not clarify the effect of the platform on Liu-Chiu. Therefore, before the next meeting, the CLD was divided into two parts, one concerning Liu-Chiu and the other concerning the platform, and the interaction between these two areas was analyzed.

**Figure 4:**
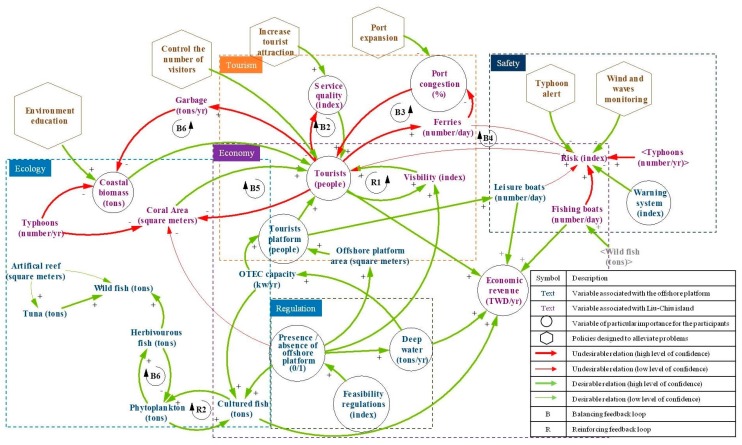
Causal-loop diagram.

The fourth meeting led to the finalization of the CLD. Shown on Figure 4, the CLD displays variables related to the platform itself in blue and variables related to Liu-Chiu in purple. Variables enclosed in circles were selected by the participants as of particular importance to them. Additionally, the units of the variables were defined and policies were formulated to alleviate potential problems (hexagons on Figure 4).

At the fifth meeting, the validity of the CLD was verified to confirm its consistency with the mental models of the participants. We asked every participant to silently go through each relation and check for something they didn’t understand and/or agree with. After five minutes, we asked them individually to state potential problems. All stakeholders replied they were satisfied with the CLD shown on Figure 4. This is not surprising since every modification of the CLD was discussed and validated by the group before it was implemented. We then qualified the relations according to two aspects: their desirability (does it trigger a desirable change?) and the degree of likeliness of the relations (are we confident this is going to happen?). Desirable (and non-desirable) relations were marked with green (red) arrows. For example, in loop B5, the positive relation between coral area and the number of tourists was deemed desirable since an increase in coral area triggers an increase in the number of tourists and both changes are good for Liu-Chiu’s community. However, the negative relation between tourist number and coral area was qualified as non-desirable since the increase in tourists would generate coral damage. Relations confirmed with a high degree of confidence are indicated by thick arrows (e.g., thick green arrow between the number of tourists and the economic revenue), and those that were more hypothetical are indicated by thin arrows (e.g., thin red arrow between the level of risk and the number of tourists). This color-coded supplementary information on the CLD allowed the stakeholders to quickly identify loops where policies were most needed and to discriminate between courses of events according to their likeliness to happen. The CLD represents a consensual mental model of the participants that was further applied to provide recommendations regarding the multipurpose offshore platform.

## 4. Results and Discussion

### 4.1. Systems Thinking Analysis

According to the stakeholders, if the multipurpose offshore platform is opened for tourism, the visibility of Liu-Chiu would increase, thereby strengthening feedback loop R1 and promote the development of tourism in Liu-Chiu. However, this would also trigger negative feedback loops B1, B2, B3, B4, and B5, all of which tend to slow down/stop the increase of tourists.

In the tourism subsystem, B2 indicates that increasing the number of tourists would undermine the quality of the services in Liu-Chiu, such as lodging and scenic areas, subsequently limiting the economic potential of Liu-Chiu. B3 reveals that increasing the number of tourists would directly affect traffic between Liu-Chiu and the main island of Taiwan; the number of trips would increase because of more tourists visiting the platform, causing the capacity of the port to become saturated, and thus limiting port development as well as the number of tourists coming to Liu-Chiu.

B1 and B5 indicate a strong relation between ecology and tourism, and the stakeholders expressed particular concern over the ecological problem. The natural environment of Liu-Chiu is the primary driver of its tourism. As indicated by B1, attracting a large number of tourists to Liu-Chiu would generate a large amount of waste and damage the island ecology. Furthermore, the stakeholders indicated that the structure of the offshore platform might be damaged by typhoons, which might be a threat to the coral ecosystem; as revealed by B5, the decrease in the coral coverage area would lower tourism on the island.

The stakeholders agreed that the multipurpose offshore platform would enhance the fishery ecology in the seas near Liu-Chiu. The integrated multi-trophic aquaculture involves using cultured phytoplankton as feed for cultured fish, thereby forming positive feedback loop R2. The cultured phytoplankton would attract more herbivorous fish to the area, increasing consumption and generating negative feedback loop B6. Increasing the population of herbivorous fish would attract more carnivorous fish to the area, causing a significant increase in the overall number of fish while increasing the wild catch yield. Both the aquaculture and the wild catch yield would benefit Liu-Chiu economically.

Finally, B4 reveals that an increase in the number of tourists would increase the carrier and recreational boat activity near Liu-Chiu; in addition, increasing the amount of wild catch would increase the number of active fishing boats in the area. Excessive boat activities would increase the marine navigation risk near Liu-Chiu, which could reduce the number of tourists. However, compared with other feedback loops, the stakeholders expressed more uncertainty on the likelihood of B4 occurring, suggesting that although safety remains one of their primary concerns, it wasn’t clear to them if it would have a critical impact on tourism.

### 4.2. Policy Analysis

The systems thinking process of developing the CLD helped examine the effects of the multipurpose offshore platform on Liu-Chiu. It revealed that all subsystems interacted together and that these interactions may generate several potential problems. Thanks to the color-coded CLD, stakeholders were able to identify potential problems and to formulate corresponding solutions, thereby providing managers and designers with a reference for developing multipurpose offshore platforms in the future. We found this was very useful for policy analysis.

If the multipurpose offshore platform is constructed, loop R1 would cause an increase in the number of tourists, but the effects of B1 and B5 would increase over time, exacerbating damage to the ecological environment and subsequently limiting the number of tourists. To mitigate this system behavior that leads to an intermediate level of tourism and a degraded environment, the stakeholders proposed two management measures. One strategy involves directly controlling the total number of tourists, thereby minimizing the effects of B1 and B5 and preserving the ecological environment in Liu-Chiu. The other strategy seeks to promote environmental education to raise tourist awareness for marine ecology protection, thus reducing the damage on the environment from B1. Implementing these two solutions would assist in safeguarding the quality of the ecological environment while still boosting the local economy.

Stakeholders also suggested upgrading the tourism industry to mitigate the effects of B2 and B3. As indicated by B2, an excessive number of tourists at a single scenic spot can reduce the quality of tourism services. Therefore, developing additional scenic spots could prevent tourists from concentrating in certain areas. B3 shows that the ports and ferries lack the carrying capacity to service a significant increase in tourist numbers. Accordingly, the stakeholders proposed expanding the ports to improve their carrying capacity and accommodate more tourists visiting the platform.

Finally, constructing the platform would increase the number of active boats such as ferries (due to B4), fishing boats (due to the increase in the amount of wild catch), and recreational boats (due to tourists visiting the platform), and subsequently increase the marine navigation risk near Liu-Chiu. According to the stakeholders, the climate and sea conditions also affect such risks. Therefore, they suggested establishing a warning system to reduce such risks. With such system, administrators could promptly receive typhoon and storm warnings and halt all sea activities when dangerous conditions are met. In addition, stakeholders indicated that the warning system should be installed on the main platform structure as well to prevent boat collisions and improve navigation safety. Stakeholders didn’t particularly stress the feedback effect of maritime safety on the number of tourists since it was deemed more uncertain.

### 4.3. Systemic Reflection

The stakeholders’ initial opinions regarding the multipurpose offshore platform changed after the GMB process. Their study of the CLD greatly improved their understanding of systemic effects, and as a result, their explanation of potential effects became more complex. Table 4 lists the differences in opinions of the stakeholders from before and after the GMB activity. Originally, the stakeholders stated that constructing the platform would benefit tourism in Liu-Chiu and that only fisheries and transportation would be negatively affected. However, after applying the systems thinking approach to construct the CLD, the stakeholders realized that increasing the number of tourists would overload local tourism resources and degrade the ecosystem of Liu-Chiu and that countermeasures were necessary.

The GMB approach helped in the identification of indirect effects (i.e., environmental degradation by tourism), limits to growth (i.e., tourism cannot grow forever without generating side-effects that will ultimately prevent it from growing more) and the realization that chronic disturbances may be more problematic than acute disturbances (i.e., the daily increase in boat traffic would generate more problems than typhoons and is easier to manage).

## 5. Conclusions

Overall, the stakeholders participating in this study approved constructing the multipurpose offshore platform in Liu-Chiu. They indicated that this would enhance local tourism, and the platform’s additional features (e.g., the deep ocean water processed through OTEC and the cage aquaculture) would stimulate the economic development in Liu-Chiu. However, the stakeholders were aware of the potential negative impacts of the project. For example, increasing the number of tourists could damage the ecosystem on the island and cause shortages in the tourism resources and service facilities, and frequent boat activities could induce maritime navigation risks. Therefore, policies and countermeasures were proposed to mitigate these impacts on Liu-Chiu.

The changes in the stakeholders’ initial opinions after constructing the CLD revealed that the GMB process helped the stakeholders better understand the system structure through systems thinking and identify how systemic problems emerged through the interactions of factors.

Although the study participants represented the opinions and interests of some of Liu-Chiu residents, they did not represent all dimensions related to the problem. Before the project is executed, the method applied in this study should be further investigated by incorporating additional dimensions to include more diverse opinions and increase the scope and depth of the analysis. Thus, numerous potential problems could be avoided when the project is initiated, and the likelihood of conflict between developers and local residents during and after project completion can be reduced. In this study, the SD model could not be constructed because of insufficient data for conducting system simulation. Collecting more data as they become available would facilitate developing a SD model for quantitative policy simulations in future studies. By doing so, decision-makers could design simulation scenarios allowing them to formulate sustainable policies.

## Figures and Tables

**Table 1:** Group Model Building (GMB) procedures.

Agenda	Topic	Contents	Model Building Result
First and second group meetings	Project description and problem articulation	Select a new location for the offshore platform, core topics and influential factors	The opinions of the chief, cage aquaculture workers, and the Dapeng Bay NSA were archived, and a preliminary CLD was created.
Third group meeting	Preliminary CLD discussion	Verify the consistency of the CLD with the stakeholders’ opinions. Verify the indices and quantified units	The CLD was revised according to the questions and opinions of the stakeholders and implemented using the indices and quantified units.
Fourth group meeting	Policy scenarios	Discuss applicable policies and scenarios	The policies proposed in the meeting were added to the CLD.
Fifth group meeting	CLD validity verification	Verify the relations among the influential factors	The CLD was modified to indicate the degree of uncertainty and desirability of influences among factors, and to verify their consistency with the mental models of the stakeholders.

Note: CLD: Causal Loop Diagram; NSA: National Scenic Area.

**Table 2:** List of the topics.

Topic	Content
Ecology	Restoring tuna, coral, and large fish populations
Economy	Wild fisheries, deep ocean water, cage aquaculture
Tourism	Sea-based bed and breakfast
Platform Design	Hydrological conditions (water temperature, depth, flow speed and direction, maximal wave height), life support pipelines, waste disposal
Safety	Effect of the climate, navigation safety
Regulations	Territorial water jurisdiction, autonomous regulation of offshore platforms in Taiwan, range of fishery rights

**Table 3:** Topics and influential factors regarding the offshore platform.

Topic	Variables	
Ecology	★ Fish population	Artificial reefs
	★ Farmed fish	Tuna
	Phytoplankton	★ Corals
Tourism	Number of rooms on the platform	Recreational boats
	★ Number of tourists	
Economy	Economic output	★ Deep ocean water
Regulations	★ Platform location	
Safety	Platform area	Warning systems
	Carriers	★ Typhoons
	Aquaculture boats	★ Risks

Note: ★ denotes variables of primary interest to the participants.

**Table 4:** Comparison of the stakeholders’ opinions before and after GMB.

Topic	Before GMB	After GMB
Ecology	Constructing the offshore platform would have only a minor effect on the ecosystem of Liu-Chiu.	The platform itself may have minor effects on the ecosystem, but the increase of tourists would generate more wastes and damages to the coastal ecosystem. Tourism regulation and education are needed.
Tourism	Constructing the offshore platform would benefit tourism in Liu-Chiu and increase the number of scenic locations.	The increase of tourists will come to an end when local tourism resources and basic facilities are overloaded. Tourism management is needed to sustainably enhance tourism.
Economy	Constructing the offshore platform would have a negative impact on the amount of wild catch.	Aquaculture cages in the platform may actually improve the amount of wild catch.
Safety	The platform structure might be prone to typhoon damage, and the subsequent wreckage might pollute the waters near Liu-Chiu and compromise navigation safety.	The wreckage scenario is a one-time event, less likely to be problematic than the increasing activity of fishing boats. Navigation safety must therefore be monitored closely and platform warning systems must be appropriately installed and operated to prevent accidents.
